# Prospective Multicenter Evaluation of [^11^C]Methionine PET/MRI Sensitivity Compared with MRI for Localizing Small Pituitary Neuroendocrine Tumor or Pituitary Adenoma in Cushing Disease

**DOI:** 10.2967/jnumed.124.269392

**Published:** 2025-10

**Authors:** Anthime Flaus, Elise Levigoureux, Julie Haesebaert, Claire Briet, Frederic Castinetti, Justine Cristante, Delphine Drui, Natacha Germain, Luigi Maione, Frederic Illouz, Emmanuel Sonnet, Igor Tauveron, Ines Merida, Sophie Lancelot, Nicolas Costes, Alexandre Vasiljevic, Lucien Marchand, Sylvie Rode, Mireille Bertholon-Gregoire, Guillaume Criton, Véronique Lapras, François Cotton, Emmanuel Jouanneau, Claire Bournaud, Gerald Raverot

**Affiliations:** 1 Nuclear Medicine Department, Hospices Civils de Lyon, Lyon, France;; 2 Lyon Neuroscience Research Center, CNRS UMR5292, INSERM, U1028, Université Claude Bernard Lyon 1, Lyon, France;; 3 Radiopharmacy Department, Hospices Civils de Lyon, Groupement Hospitalier Est, Bron, France;; 4 Research on Healthcare Performance, INSERM, Université Claude Bernard Lyon 1, Lyon, France;; 5 Pôle de Santé Publique, Service de Recherche et d’Épidémiologie Cliniques, Hospices Civils de Lyon, Lyon, France;; 6 Department of Endocrinology, Reference Center for Rare Pituitary Diseases, Angers University Hospital, Angers, France;; 7 CarMe team, MITOVASC, CNRS UMR6015, INSERM, U1083, Angers University, Angers, France;; 8 Marseille Medical Genetics, INSERM, UMR1251, Institut MarMaRa, Department of Endocrinology, Reference Center for Rare Pituitary Diseases, Hôpital La Conception APHM, Aix Marseille University, Marseille, France;; 9 IRIG Biosanté, INSERM, UMR1292, CEA, CHU Grenoble Alpes, University Grenoble Alpes, Grenoble, France;; 10 Department of Endocrinology Diabetology and Nutrition, L’institut du Thorax, CHU de Nantes, Nantes University, Nantes, France;; 11 Department of Endocrinology, Diabetes, Metabolism and Eating Disorders, University Hospital of Saint-Etienne, Saint-Etienne, France;; 12 TAPE Research Group, Eating Disorders, Addictions & Extreme Bodyweight, Jean Monnet University, Saint-Etienne, France;; 13 Physiologie et Physiopathologie Endocriniennes, INSERM, UMR-S1185, Service d’Endocrinologie et des Maladies de la Reproduction, Hôpital Bicêtre APHP, Université Paris-Saclay, Le Kremlin-Bicêtre, France;; 14 Service Diabétologie–Endocrinologie, CHRU Brest, Brest, France;; 15 Service d’Endocrinologie, Diabétologie et Maladies Métaboliques, CHU Clermont-Ferrand, Clermont-Ferrand, France;; 16 Laboratoire GReD, Université Clermont Auvergne, Clermont-Ferrand, France;; 17 CERMEP-Life Imaging, Lyon, France;; 18 Department of Neuropathology, Hospices Civils de Lyon, Bron, France;; 19 Service d’Endocrinologie, Hôpital Saint Joseph Saint Luc, Lyon, France;; 20 Centre de Consultation de la Sauvegarde, Lyon, France;; 21 Clinique du Renaison, Roanne, France;; 22 Service de Radiologie, Centre Hospitalier Lyon Sud, Hospices Civils de Lyon, Lyon, France;; 23 Université Claude Bernard Lyon 1, Villeurbann, France;; 24 Laboratoire CREATIS, CNRS UMR5220, INSERM, U1296, INSA Lyon, Villeurbanne, France;; 25 Skull Base and Pituitary Unit, Department of Neurosurgery B, Neurological Hospital, Lyon, France;; 26 CNRS UMR5286, INSERM, U1052, Cancer Research Center of Lyon, Lyon, France;; 27 Service d’Endocrinologie, Centre Hospitalier de Roanne, Roanne, France; and; 28 Endocrinology Department, Reference Center for Rare Pituitary Diseases, Hospices Civils de Lyon, Groupement Hospitalier Est, Bron, France

**Keywords:** PET/MRI, [^11^C]methionine, amino acid, brain, corticotroph

## Abstract

Functional small pituitary neuroendocrine tumors (PitNETs) of the corticotroph type lead to Cushing disease (CD), a condition associated with significant morbidity and increased mortality. Pituitary MRI is the primary imaging modality used to localize the tumor, but it is inconclusive in up to 30% of cases. Accumulating retrospective evidence suggests that [^11^C]methionine ([^11^C]MET) PET could address this diagnostic gap. This prospective study evaluates the sensitivity of [^11^C]MET PET/MRI compared with MRI for small PitNET localization in CD. **Methods:** This prospective multicenter study (ClinicalTrials.gov NCT03346954) included consecutive patients with biochemically confirmed de novo CD who underwent [^11^C]MET PET/MRI before surgery. Images were evaluated by experienced radiologists and nuclear medicine physicians. Their sensitivity to correctly localized PitNETs was calculated, with pathologic analysis of the surgical specimen used as a reference. **Results:** Thirty patients (73% women; mean age, 39.4 ± 12.7 y) underwent PET/MRI, and pathology confirmed PitNET in 22 patients (73%). [^11^C]MET PET/MRI correctly localized the tumor in 18 patients (82%; 95% CI, 0.60–0.95), whereas MRI correctly localized the tumor in 19 patients (86%; 95% CI, 0.65–0.97; *P* = 1.0). [^11^C]MET PET/MRI and MRI were concordant in 18 patients (82%), with the tumor correctly localized in 17 of 18 patients (94%). Among the 4 patients with discordant findings, MRI correctly localized the tumor in 2 cases for which PET was inaccurate; in 1 case, PET accurately identified the lesion, which was not visualized on MRI, and in the final case, both modalities failed to localize the tumor. **Conclusion:** This prospective study shows that [^11^C]MET PET/MRI has high sensitivity but does not differ significantly from 3-T MRI for the accurate localization of small PitNETs of the corticotroph type in patients with de novo CD. Future research could benefit from emerging imaging technologies and should focus on optimizing PET imaging protocols and exploring alternative radiotracers.

## Visual Abstract

**Figure:**
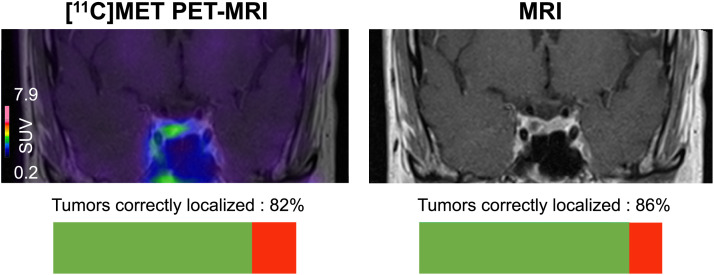

Cushing disease (CD) is a rare endocrine disorder (1) characterized by excessive secretion of adrenocorticotropic hormone (ACTH) from a pituitary neuroendocrine tumor (PitNET) or pituitary adenoma of the corticotroph type (2), leading to chronic hypercortisolism. This condition significantly affects morbidity and mortality (3). Multidisciplinary clinical management typically involves transsphenoidal surgery as first-line therapy, which is facilitated by accurate preoperative tumor localization (4). Although the impact of preoperative target absence on surgical outcomes is debated (5), accurate preoperative tumor localization could lead to better surgical outcomes (6,7).

MRI with a dedicated pituitary protocol, despite its limitations (8), is the primary imaging diagnostic method. Its sensitivity is approximately 70% for corticotroph-type PitNETs (9), mainly because more than 90% of these tumors are small (<10 mm in diameter), which leads to false-negative results (10,11). In addition, nonfunctioning PitNETs, which are present in up to 10% of the population (12,13), as well as MRI artifacts, can result in false-positive results. Consequently, in cases of suspected small PitNET on MRI, there is a consensus that bilateral inferior petrosal sinus sampling (BIPSS) should be performed to confirm the central origin of the hypercortisolism, despite its invasiveness, cost, risk of complications, and low accuracy for tumor lateralization (4,11,13).

To overcome the limitations of current imaging techniques, molecular imaging, particularly PET, offers a promising noninvasive alternative. Various radiopharmaceuticals have been explored, mainly [^18^F]FDG PET and somatostatin receptor PET imaging, but these have shown insufficient sensitivity (14–17). In contrast, there is accumulating retrospective evidence that PET imaging using radiolabeled amino acids may present added value in this setting (18,19). The rationale for using radiolabeled amino acids is based on the overexpression of L-type amino acid transporters (20) and the high secretory activity of PitNETs, although the precise relationship between amino acid uptake and secretory activity remains unclear (21). Prior studies evaluating [^11^C]methionine ([^11^C]MET) PET have reported sensitivities ranging from 70% to 100% for corticotroph-type PitNETs (18,22–24). Nevertheless, 1 retrospective study emphasized limitations inherent to standard visual analysis, highlighting difficulties in distinguishing small PitNETs from physiologic background uptake (22). This observation suggests that despite promising sensitivity, [^11^C]MET PET might not consistently offer sufficient sensitivity to accurately localize small PitNETs.

The present study thus aimed to prospectively evaluate the sensitivity of [^11^C]MET PET/MRI for the correct localization of small PitNETs in de novo CD and compare this sensitivity to that of MRI, using pathologic analysis of the surgical specimen as a reference.

## MATERIALS AND METHODS

### Study Design and Patients

This prospective multicenter cohort study (ClinicalTrials.gov NCT03346954) recruited patients from 5 centers in France. Patients were included if the following criteria were met: 18 y of age or older, biochemically proven diagnosis of ACTH-dependent de novo CD, completion of the usual work-up to confirm the central origin of hypercortisolism (positive pituitary MRI or BIPSS in cases of inconclusive or negative MRI, according to French guidelines) (25), and indication for transsphenoidal surgery. Patients were excluded if any of the following criteria was met: macroadenoma on MRI, recurrent CD, or prior pituitary surgery.

The study was performed in line with the principles of the Declaration of Helsinki and was approved by the Ile de France VIII Ethics Committee (EudraCT 2017-002721-38). Written informed consent was obtained from all patients before data collection. The Standards for Reporting of Diagnostic Accuracy Studies checklist (26) was followed.

### Patient Management and Data Collection

Patients was managed in their respective centers according to standard local practices. After inclusion, all patients had [^11^C]MET PET/MRI performed at Hospices Civils de Lyon before undergoing transsphenoidal surgery. PET results did not influence patient management, because the surgeons and clinicians in charge were masked to the results.

Demographic data (age and sex); medical treatment; BIPSS results; tumor secretory activity, as indicated by ACTH serum level and 24-h urinary free cortisol (UFC); surgical localization (left, right, or median); pathologic results (PitNET positive or negative); Ki-67 proliferation index; and postoperative biochemical status, as indicated by plasma ACTH and cortisol level 24 h after the surgery, were collected from medical records. Pathologic analysis and hormone secretion assessment were conducted locally at each participating center according to standard clinical practice (supplemental materials [supplemental materials are available at http://jnm.snmjournals.org]).

### Image Acquisition

Imaging was performed on a 3.0-T PET/MRI hybrid scanner (Biograph mMR; Siemens). After an intravenous bolus injection of 337 ± 80 MBq of [^11^C]MET, PET data acquired between 20 and 40 min after injection were used for reconstruction. The acquired MRI sequences were T1-weighted magnetization prepared–rapid gradient echo, T2-weighted time spin echo coronal, T1-weighted time spin echo coronal and sagittal (before and after gadolinium injection), and dynamic T1-weighted time spin echo coronal. Details with regard to [^11^C]MET synthesis, PET reconstruction and data correction, and MRI sequences are presented in the supplemental materials.

### Image Analysis

All imaging analyses were performed by experienced readers masked to the clinical data. MR images obtained from PET/MRI were reviewed by a single radiologist; in cases of discordance with the initial MRI performed during routine management, a second experienced radiologist reviewed the MRI and a consensus was reached. Localization was categorized as left, right, or median in the coronal plane. Both radiologists measured the tumor in 3 planes to compute its volume by the formula proposed by Lundin and Pedersen (27).

The joint analysis of PET and MR images was performed by a nuclear medicine physician and a radiologist, and a consensus was reached when necessary. PET and MR images were analyzed using Syngo.via software (Siemens). If the PET and MR images were concordant and indicated PitNET presence, that is, uptake higher than background pituitary uptake (28), localization was categorized as left, right, or median. When PET and MRI results were discordant, PET-alone localization was categorized as left, right, or median. Subsequently, a semiquantitative analysis was performed to determine the SUVs of the suspected PitNET and of normal brain parenchyma. Tumor-to-background ratios (TBRs) were calculated based on these values, using guidelines adapted from glial tumors, as detailed in the supplemental materials (29).

### Study Endpoints

The primary endpoint was the sensitivity of [^11^C]MET PET/MRI for correctly localizing small PitNET using the localization of the surgical specimen as a reference, after pathologic confirmation of PitNET of the corticotroph type. [^11^C]MET PET/MRI sensitivity was then compared with that of MRI for correctly localizing small PitNET. Patients without pathologic confirmation were excluded from the primary endpoint analysis. In cases of discordant PET/MRI findings, patients were classified as negative in all but 1 instance, in which PET was positive and MRI was negative, to conservatively account for the added diagnostic value of combined imaging.

The secondary endpoints were image analysis results for the entire cohort (i.e., including patients without pathologic confirmation), comparison of patient characteristics between correctly and incorrectly localized PitNETs by PET/MRI, assessment of potential associations between tumor characteristics (secretory activity and Ki-67 index) and [^11^C]MET uptake TBR for PitNETs correctly localized by PET/MRI, and description of postoperative biochemical status.

### Statistical Analysis

Continuous variables were reported by mean ± SD for normally distributed variables and by median and interquartile range for nonnormally distributed variables. Categoric variables were reported as frequencies and percentages.

PET/MRI and MRI sensitivities were calculated against pathologic analysis as a reference. Two-sided 95% CIs were computed using an exact binomial test. Imaging results from PET/MRI and MRI were compared using an exact McNemar test. The comparison of patient characteristics between correctly and incorrectly localized PitNETs by PET/MRI was performed using the χ^2^ or Fisher exact test for categoric data and the Mann–Whitney *U* test for continuous data because of the limited sample size. Correlations between tumor characteristics and [^11^C]MET uptake were assessed using the Pearson correlation coefficient.

### Power Analysis

Given the rarity of CD, feasibility dictated patient inclusion. Assumptions for the calculation of the sample size included a predicted 90% positive [^11^C]MET PET/MRI rate (18,22–24) versus 70% for MRI (9). The targeted sample size of 30 participants provided a statistical power of 76% to detect differences in localization rates with a 1-sided α-risk of 5%. Adjusting for 10% dropout or nonevaluable cases, the final sample size was set at 33 patients.

## RESULTS

### Patients

**FIGURE 1.**
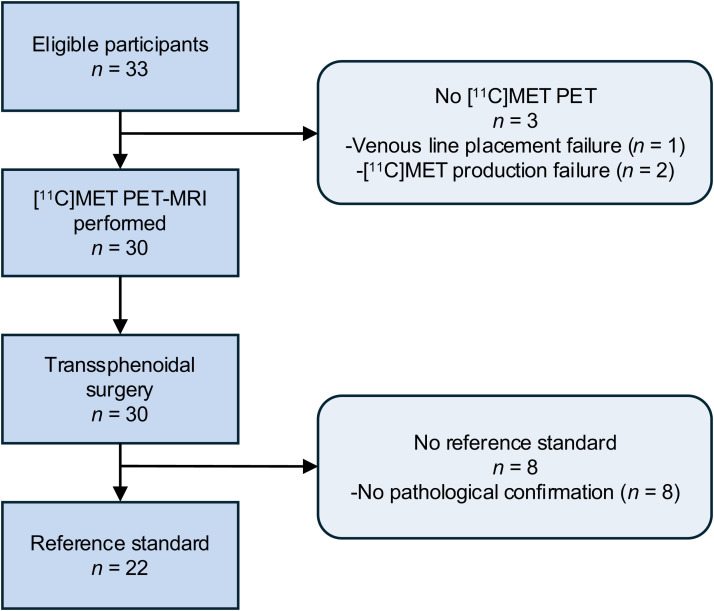
Flow diagram of patient inclusion.

Thirty-three patients were recruited between December 2017 and July 2020, 3 of whom were withdrawn due to venous line placement failure in 1 and lack of [^11^C]MET injection because of production failure in 2 (Fig. 1). Among the 30 patients who completed the PET/MRI, no adverse events occurred. BIPSS was performed in 5 patients (17%). Overall, 22 patients were women (73%), mean age was 39.4 ± 12.7 y, and mean weight was 85.4 ± 20.8 kg. At inclusion, median 24-h UFC was 3 (interquartile range, 1.8–5.9) times above the upper limit of normal and mean ACTH was 1.2 ± 0.97 times above the upper limit of normal (Supplemental Table 1). Among all patients, 8 (27%) were on medical treatment (adrenal steroidogenesis inhibitors) before surgery: ketoconazole for 5 (17%), mitotane for 1 (3%), osilodrostat for 1 (3%), and metyrapone for 1 (3%). Surgery was performed in all 30 patients at a median of 21 d after PET/MRI (interquartile range, 8–51.3), and 22 patients (73%) had a pathologically confirmed PitNET of the corticotroph type. Tumor localization according to the surgeon is described in Supplemental Table 2.

**FIGURE 2.**
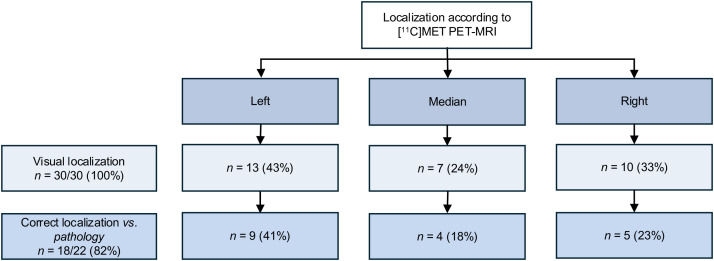
PitNET localization according to [^11^C]MET PET/MRI.

**FIGURE 3.**
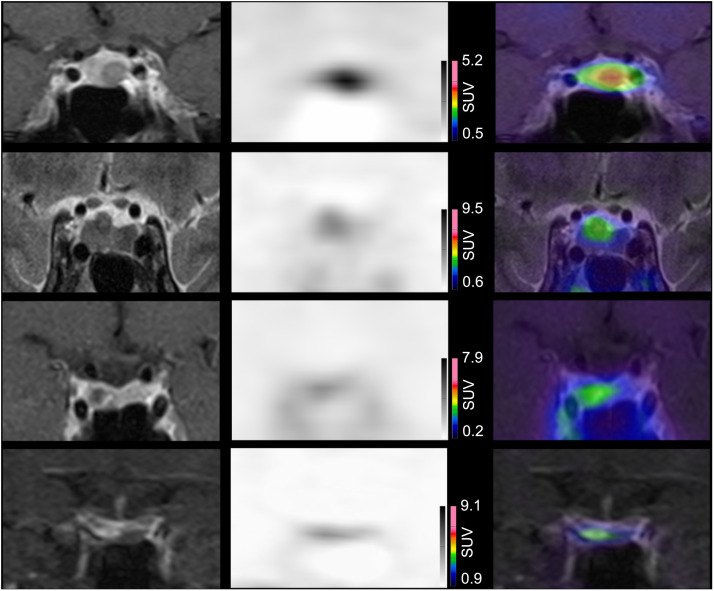
First column shows coronal MR images (first and third rows, T1-weighted time spin echo [TSE] after gadolinium MRI; second row, T2-weighted TSE; fourth row, dynamic T1-weighted TSE). Second column displays corresponding [^11^C]MET PET images. Third column presents superimposed PET and MR images. First row: Patient 4 shows clear left-sided focal uptake, concordant with 8-mm tumor identified on MRI and confirmed by pathology. Second row: Patient 11 shows clear median focal uptake, concordant with 5-mm tumor on MRI and confirmed by pathology. Third row: Patient 29 shows clear right-sided focal uptake, concordant with 6-mm tumor on MRI and pathologic findings. Fourth row: Patient 15 shows right-sided focal uptake not matching 5-mm tumor observed on MRI, and both findings are discordant with pathology.

Among the 22 patients with a pathologically confirmed PitNET, [^11^C]MET PET/MRI correctly localized the tumor in 18 cases (82%; 95% CI, 0.60–0.95; Fig. 2), whereas MRI correctly localized the tumor in 19 cases (86%; 95% CI, 0.65–0.97; *P* = 1.0; examples are in Fig. 3). [^11^C]MET PET/MRI and MRI were concordant in 18 patients (82%), with the tumor correctly localized in 17 of 18 patients (94%). Among the 4 patients with discordant PET/MRI and MRI findings—all of whom were classified as being in remission—MRI correctly localized the tumor in 2 cases for which PET was inaccurate; in 1 case, PET accurately identified the lesion, which was not visualized on MRI, and in the final case, both modalities failed to localize the tumor.

### Secondary Endpoints

In the whole cohort, MRI suggested the localization of a PitNET in 28 of 30 patients (93%), whereas PET/MRI suggested the localization of a PitNET in 30 of 30 patients (80%; Supplemental Table 2). Among patients with a pathologically confirmed PitNET, the subgroup comparison between correctly and incorrectly localized PitNETs using [^11^C]MET PET/MRI (*n* = 18 and *n* = 4, respectively) found no significant difference in terms of age, sex, BIPSS performed, tumor volume, TBR, Ki-67 index, ACTH serum level, and use of medical treatment (Table 1). In addition, in the subgroup of patients with correctly localized PitNETs using [^11^C]MET PET/MRI, no correlation between TBR and ACTH serum levels (ρ = −0.1, *P* = 0.7), 24-h UFC (ρ = −0.14, *P* = 0.6), and Ki-67 (ρ = 0.06, *P* = 0.8) was found (the correlation plot is shown in Supplemental Fig. 1).

**TABLE 1.** Characteristics of Patients According to Correct or Incorrect Localization of PitNET by [^11^C]MET PET/MRI

Characteristic	Correctly localized with PET/MRI	Incorrectly localized with PET/MRI	*P*
*n*	18	4	
Tumor volume (mm^3^)	102.9 ± 80.4	66.1 ± 43.1	0.59
TBR	3.2 ± 0.6	3.3 ± 1.3	0.97
Women*	12 (67%)	4 (100%)	0.54
BIPSS*	2 (11%)	2 (50%)	0.13
Age (y)	42.8 ± 12.3	35.3 ± 14.2	0.37
UFC (×ULN)^†^	3 (1.6–4.3)	4.5 (1.9–7.7)	0.59
ACTH (×ULN)	1.2 ± 0.9	1.3 ± 0.7	0.65
Medical treatment*	7 (39%)	1 (25%)	0.07
Ki-67 index (%)	2.8 ± 2.3	3.0 ± 4.8	0.30

* Data are number and percentage of patients.

† Data are median and interquartile range.

×ULN = times above upper limit of normal.

Data are expressed as mean ± SD unless otherwise specified.

Among all patients, 26 were considered to be in postoperative biochemical remission. The 22 patients with a pathologically confirmed PitNET were considered to be in remission: 18 (82%) with postsurgical corticotroph insufficiency and 4 (18%) with normal corticotroph function. Among the 8 patients (27%) with no pathologic confirmation of PitNET, 4 (50%) had persistent disease, 2 (25%) had normal corticotroph function, and 2 (25%) had corticotroph insufficiency (Supplemental Table 2).

## DISCUSSION

To our knowledge, the present study offers the first prospective evaluation of [^11^C]MET PET/MRI for localizing small PitNETs in newly diagnosed CD, directly comparing its performance with MRI. The findings indicate that [^11^C]MET PET/MRI has a high sensitivity of 82% that does not statistically outperform the 3-T MRI sensitivity of 86% for correct localization.

The proportion of accurate PitNET localization using PET/MRI in the present study was comparable to the 70%–100% range previously reported in smaller retrospective studies, which included at most 12 patients (18,22–24). The comparison of the present findings with these previous studies is limited due to differences in study designs. Moreover, most previous studies focused on detection rather than precise localization. Two previous retrospective studies reported 100% positivity of [^11^C]MET PET but lacked detailed correlation with intraoperative findings and postoperative outcomes (23,24). Another retrospective study showed 88% positivity for [^11^C]MET PET, but lateralization was not analyzed visually; PET images were directly compared with the surgeon’s intraoperative observations before being confirmed by pathologic analysis (18). Nevertheless, the findings herein, which are more robust due to the larger sample size and prospective design, suggest that [^11^C]MET PET/MRI may provide additional value compared with MRI alone for the accurate localization of small PitNETs in de novo CD.

The present findings highlight the performance of 3-T MRI, which correctly localized small adenomas in 86% of the cases, a proportion slightly higher than the 71% mean reported in a recent literature review (9). This could be explained partly by the involvement of a third reader in cases of discrepancy between the first readers, which occurred in 20% of the cases herein, underlining the value of multiple readings from experienced radiologists.

Overall, approximately two tenths of the included patients had incorrect small PitNET localization, highlighting the need for further improvement. Both MRI and molecular imaging show promise in this regard. For instance, [^18^F]fluoroethyl-l-tyrosine, another amino acid PET tracer, is gathering interest because of its longer half-life (109.7 min for ^18^F vs. 20.4 min for ^11^C), allowing a wider availability (29,30). Two recent retrospective cohorts demonstrated the high accuracy of [^18^F]fluoroethyl-l-tyrosine PET in localizing corticotroph-type PitNETs, showing improvement over BIPSS and [^11^C]MET (18,31). Unlike [^11^C]MET, [^18^F]fluoroethyl-l-tyrosine appears to have lower background uptake in the pituitary (18,31,32). Another innovative radiotracer consists of the in vivo detection of corticotropin-releasing hormone 1 receptor expression in the pituitary gland. A study on corticotropin-releasing hormone PET demonstrated 100% accuracy compared with pathologic findings in a cohort of 14 patients (33). Furthermore, advances in PET hardware, such as brain-dedicated PET systems, have shown improved resolution and contrast recovery, offering the potential to improve detection of small lesions (34). MRI technology is also evolving, with ultra–high-field (7 T) MRI becoming more clinically available. It offers improved signal-to-noise and contrast-to-noise ratios compared with conventional 1.5- or 3-T MRI (35). Although it has the potential to improve lesion detection, achieving perfect sensitivity and specificity remains challenging because of increased field inhomogeneities, which could potentially lead to more incidental findings (36).

As a functional imaging modality, an additional potential benefit of [^11^C]MET PET lies in its ability to provide information about the secretory activity of PitNETs, given that amino acids are essential for synthesizing the secreted proteins. Although the ACTH serum level directly reflects the secretory activity of PitNET, and thus its need by amino acids for protein synthesis, 24-h UFC can be considered an indirect marker of daily averaged ACTH secretion. However, in our study, neither ACTH serum levels nor 24-h UFC values showed a correlation with [^11^C]MET uptake. The lack of correlation with 24-h UFC values is consistent with findings from a previous study (21). This may be explained by the inclusion, in both studies, of patients receiving adrenal steroidogenesis inhibitors, which are intended to normalize UFC levels. The results suggest the need for treatment-specific studies to assess the impact of adrenal steroidogenesis inhibitors on PitNET [^11^C]MET PET uptake. With regard to ACTH serum levels, the lack of correlation could have occurred because ACTH was not measured at the time of PET imaging. Considering ACTH’s short half-life (∼15 min) and its highly variable secretory pattern (37), the timing of measurement may be critical for establishing any relationship. Lastly, no trend between Ki-67, a proliferation index, and TBR was observed, suggesting that [^11^C]MET PET may not provide insights into tumor proliferation (38), although this warrants further investigation.

To our knowledge, the present study is the first prospective evaluation of [^11^C]MET PET/MRI for localizing small PitNETs in patients with de novo CD. Despite the relatively small sample size, this remains the largest study to date, reflecting the rarity of the disease. In addition, the absence of pathologic confirmation in 8 patients underscores the inherent challenges in definitively localizing corticotroph-type small PitNETs. Furthermore, the study design, inclusion criteria, and positivity of [^11^C]MET PET in all cases prevented the calculation of metrics such as specificity and negative predictive value. Finally, the added value of PET/MRI in cases of negative MRI could not be clearly evaluated herein, because most patients had a correctly localized lesion on MRI (19/22 patients). Nevertheless, according to international guidelines (4), all patients in this study would have theoretically been eligible for BIPSS. In this context, a positive PET/MRI result could offer an alternative by providing sufficient localization information to potentially bypass the need for this invasive procedure.

These limitations highlight the complexity of diagnosing small PitNETs. They also reinforce the need for studies that explore larger, multicenter cohorts and incorporate emerging imaging technologies, such as new PET tracers and ultra–high-field MRI, to address these challenges and optimize diagnostic accuracy.

## CONCLUSION

This prospective study shows that [^11^C]MET PET/MRI has high sensitivity for accurately localizing small PitNETs of the corticotroph type in patients with de novo CD but that this sensitivity is not significantly different from that of 3-T MRI. Future research should focus on exploring alternative radiotracers and could potentially benefit from emerging imaging technologies.

## DISCLOSURE

This study was financially supported by a grant from the French ministry of health (PHRCI-N-2016). No other potential conflict of interest relevant to this article was reported.

## Notes

KEY POINTS **QUESTION:** What is the sensitivity of [^11^C]MET PET/MRI compared with that of MRI for localizing small PitNETs of the corticotroph type in de novo CD? **PERTINENT FINDINGS:** In this prospective study, the 82% sensitivity of [^11^C]MET PET/MRI was not significantly different from the 86% sensitivity of MRI for localizing small PitNETs. **IMPLICATIONS FOR PATIENT CARE:** 3-T MRI remains the primary diagnostic tool, demonstrating high accuracy in this cohort. [^11^C]MET PET/MRI may have added value in certain challenging cases.
